# Predicting Co-Author Relationship in Medical Co-Authorship Networks

**DOI:** 10.1371/journal.pone.0101214

**Published:** 2014-07-03

**Authors:** Qi Yu, Chao Long, Yanhua Lv, Hongfang Shao, Peifeng He, Zhiguang Duan

**Affiliations:** 1 Department of Medical Information Management, Shanxi Medical University, Taiyuan, Shanxi, China; 2 School of Medicine, Stanford University, Stanford, California, United States of America; 3 Department of Science and Technology, Shanxi Medical University, Taiyuan, Shanxi, China; 4 School of Public Health, Shanxi Medical University, Taiyuan, Shanxi, China; University of Illinois-Chicago, United States of America

## Abstract

Research collaborations are encouraged because a synergistic effect yielding good results often appears. However, creating and organizing a strong research group is a difficult task. One of the greatest concerns of an individual researcher is locating potential collaborators whose expertise complement his best. In this paper, we propose a method that makes link predictions in co-authorship networks, where topological features between authors such as Adamic/Adar, Common Neighbors, Jaccard's Coefficient, Preferential Attachment, Katz_β_, and PropFlow may be good indicators of their future collaborations. Firstly, these topological features were systematically extracted from the network. Then, supervised models were used to learn the best weights associated with different topological features in deciding co-author relationships. Finally, we tested our models on the co-authorship networks in the research field of Coronary Artery Disease and obtained encouraging accuracy (the precision, recall, F1 score and AUC were, respectively, 0.696, 0.677, 0.671 and 0.742 for Logistic Regression, and respectively, 0.697, 0.678, 0.671 and 0.743 for SVM). This suggests that our models could be used to build and manage strong research groups.

## Introduction

Research collaborations can be conceptualized as a research effort done by research groups from either the same country or disparate countries [1]. It is widely believed that these collaborations have a synergistic effect, because the combined expertise of group members always yields results that surpass the sum of the individual's capabilities [2]. However, building and organizing such research groups is not an easy task. One of the greatest concerns of an individual researcher is how to find a suitable collaborator. Given the difficulty involved in predicting which collaborations have the greatest potential for success, experts within a specific domain are uncertain with whom they should collaborate.

This problem could be alleviated if researchers had access to the experts' research interests and ongoing research activities. This information could be used to determine these researchers' level of expertise within the field, and thus help establish whether they would serve as an appropriate collaborator of both comparable and compatible expertise. However, such information is often unavailable and difficult to obtain since no centralized sources exist.

Given a corpus of literatures, co-authorship networks can be easily constructed, with nodes representing researchers and links representing co-authorships. Topological features (such as Adamic/Adar, Common Neighbors) in co-authorship networks offer a good way of predicting future co-author relationships between existing authors [3]. In other words, if we could predict the appearance of new links between two existing authors in co-authorship networks with a reasonable accuracy, these new links then might be reasonable suggestions for potential research collaborations.

In this paper, structural topological features were extracted from the co-authorship networks, and supervised models were used to learn the best weights associated with different topological features in deciding the co-author relationships. We tested our methods on the co-authorship networks within medical research domain and the results confirmed that the appearance of co-author relationships is dependent on the network's topological structures and that supervised learning methods can help to exploit this dependence when making co-author relationship predictions.

## Literature Review

Link prediction in complex networks aims to estimate the likelihood that a link exists between two nodes, based on the observations of existing links and the attributes of the nodes.

Link prediction problems were originally solved through Markov chains. Sarukkai applied link prediction and path analysis, based on Markov chains, to web server http request predictions, adaptive web navigation, tour generation and personalized hub/authority [4]. Zhu et al. built a Markov model for link predictions of web site based on past users' visit behaviors as recorded in the web log file. A few years later, another set of link prediction algorithms, this time based on the similarity of two nodes (e.g., common neighbors), was proposed [5], [6]. Liben-Nowell and Kleinberg were the first that applied structure-based node similarity indices towards predicting links in social networks. They systematically compared several topological features, including graph shortest distance, common neighbors, preferential attachment, Adamic/Adar, Jaccard, SimRank, hitting time, rooted PageRank, and Katz_β_, to examine the link prediction problem in co-authorship networks [3]. Pavlov and Ichise test their link prediction models on a co-authorship network within the domain of Japanese electronics information and communication engineer, and obtained link predictors with encouraging accuracy [2]. Lü et al. studied nine well-known local topological features on six real networks extracted from disparate fields, as well as proposed two new local features [7]. They also applied local similarity indices to the link prediction problem in weighted networks, and found that the weak ties play a significant role in the co-authorship link prediction [8]. Meng et al. proposed semi-local indexes in both un-weighted and weighted networks by introducing the resource allocation process into the Local Path index [9]. Sun et al. studied the problem of co-author relationship prediction in the heterogeneous bibliographic network, in which there are multiple types of objects (e.g., journals, topics and authors), and proposed a new methodology called PathPredict to solve the problem [10]. More recently, Lei and Ruan presented a new link prediction model based on topological similarities measured by a novel random walk-based procedure [11].

Link prediction problems have been explored in different networks, such as web page networks [4], [5], food webs [12], protein networks [11], [13], [14], gene regulatory networks [15], adverse drug reaction networks [16], social communities networks [17], [18], co-authorship networks [2], [3], [10], [19], [20], and paper citation networks [21].

The aforementioned studies mainly based their link prediction models on a single topological feature, such as common neighbors or Admic/Adar. In this study, however, we seek to solve link prediction problems in co-authorship networks by combining several widely used topological features, and then compare these results with those generated by individual topological features. Furthermore, we applied the link prediction models to the co-authorship networks in the biomedical research domain, while previous studies mainly focused on co-author relationship prediction in the computer science research domain.

## Methodology and Data

### Topological features

Let 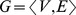 be a graph with nodes 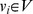 and edges 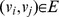, 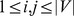. Various network topological features for each pair of nodes in the graph can be computed. These features may be correlated with the probability that a link between the nodes will appear in the future. The set of topological features for a pair of nodes forms a feature vector.

A multitude of topological features can be used for a pair of nodes according to the studies by [2] and [3]. In this paper, 5 topological features documented in both [2] and [3] were chosen for co-author relationship prediction (Table 1). We also included PropFlow, another topological feature, in this paper (Table 1), because it has been shown to outperform common neighbors, Jaccard's coefficient and Adamic/Adar [18].

**Table 1:** Formula for the 6 topological features used in this paper.

Type	Topological feature	Description
Neighborhood-based	Common Neighbors	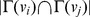
	Jaccard's coefficient	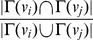
	Adamic/Adar	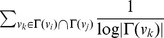
	Preferential attachment	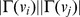
Path-based	Katz_β_	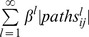 . 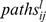 denotes the number of paths of length *s* connecting *v_i_* and *v_j_*.
	PropFlow	the probability that a restricted random walk starting at *v_i_* ends at *v_j_* in *l* steps or fewer using link weights as transition probabilities.

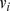
 denotes node i. 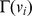 denotes the set of all neighbors of 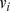. 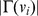 denotes the number of all neighbors of 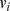.

#### Common neighbors

Common neighbors is defined as the number of common neighbors shared by two nodes *v_i_* and *v_j_*. Newman verified a correlation between the number of common neighbors of *v_i_* and *v_j_* at the time *t*, and the probability that they will collaborate in the future [22].

#### Jaccard's coefficient

Jaccard's coefficient is a normalized measure of common neighbors. It computes the ratio of common neighbors out of all neighbors, and can be used for comparing the similarity and diversity of neighbor set.

#### Adamic/Adar

Adamic/Adar, a weighted version of common neighbors, assigns greater weight to common neighbors *v_k_* of *v_i_* and *v_j_* which themselves have fewer neighbors. This means the contribution of a common neighbor to the score is weighted in proportion to the rarity of the neighbor.

#### Preferential attachment

Preferential attachment was introduced by Barabási and Albert to explain the power-law degree distribution in complex real-world networks [23]. It is defined as the product of the neighbours of *v_i_* and *v_j_*. Preferential attachment means that the more connected a node is, the more likely it is to receive new links. Nodes with higher degree have stronger ability to grab links added to the network.

#### Katz_β_

Katz_β_ defines a measure that sums over all paths between two nodes, exponentially damped by length to count short paths more heavily.

#### PropFlow

PropFlow assigns the weights to each path using the products of proportions of the flows on the edges [18]. It is it is a more localized measure of propagation, and is insensitive to topological noise far from the source node.

Lpmade, a complete cross-platform software, was used for calculating topological features in the co-authorship networks [24].


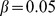
 and 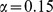 were used in the paper because they are the commonly accepted values in the research community [17], [25].

### Prediction models

We then build the relationship prediction models that model the probability of co-authorship between two authors as a function of topological features between them. In this paper, we chose the logistic regression (LR) and Support Vector Machines (SVM) as our prediction models. LR is one of the most widely used classification methods, while SVM has more recently become an important alternative.

#### LR

For each training pair of authors 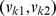, let 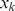
be the 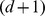-dimensional vector including constant and d topological features between them, and y_k_ be the label of whether they will be will be co-authors in the future (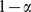 if they will be co-authors, and otherwise 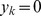), which follows binomial distribution with probability 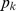. The probability 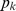 is modelled as follows:
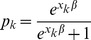



Where 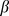 is the 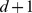 coefficient weights associated with the constant and each topological feature. We then use the standard MLE (Maximum Likelihood Estimation) to derive 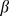, which maximizes the likelihood of all the training pairs:
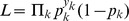



#### SVM

The basic idea of SVM is as follows: a vector containing n features can be mapped to a point in n-dimensional space (where each dimension corresponds to a feature). Thus, our author pairs can be represented by a set of points in the space. Each point then has its own binary label. The goal is to separate the points into two groups so that points with the same label are in the same group. This can be realized by using a linear separator (i.e., an n-dimensional hyperplane), which was adopted in this paper. To minimize generalization error, the hyperplane is usually chosen in such a way as to maximize the margins on both its sides. We use the sequential minimal optimization (SMO) training algorithm, since it is known to perform well with linear SVM.

Weka (Waikato Environment for Knowledge Analysis), version 3.6.9, was used to implement LR and SMO [26]. For the LR model, the default values for parameter settings were used. For the SMO model, we set buildLogisticModels as “True” in order to fit logistic models to the output and used the default values for all other parameters.

Stratified 10-fold cross-validation was used to predict the accuracy rate of the learning models above.

### Model evaluation

Let us consider classification problems using only two classes, in which the outcomes are labelled either as positive (*p*) or negative (*n*). There are four possible outcomes. If the outcome from a prediction is *p* and the actual value is also *p*, then it is counted as a true positive (*TP*); if the actual value is *n* then it is said to be a false positive (*FP*). Conversely, a true negative (*TN*) has occurred when both the prediction outcome and the actual value are *n*, and false negative (*FN*) is when the prediction outcome is *n* while the actual value is *p*. Then several metrics can be calculated. Here we introduce four of them: precision, recall, F1 score and AUC.

#### Precision

Precision is defined as the proportion of true-positive predictions out of all positive predictions. It is useful in determining how well the model fits the whole data.
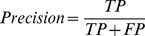



#### Recall

Recall (also called true positive rate, *tp rate*) is the proportion of true-positive predictions out of all true labels. It represents how well the model is able to predict future collaborations.
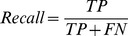



#### F1 score

F1 score (also F-score or F-measure) can be interpreted as a weighted average of the precision and recall, where an F1 score reaches its best value at 1 and worst score at 0. The traditional F1 score is the harmonic mean of precision and recall:
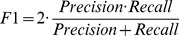



#### AUC

AUC is the area under an ROC curve. An ROC curve is a graphical plot that illustrates the performance of a binary classifier system as its discrimination threshold is varied. One more term, “false positive rate (fp)” should be introduced before we discuss ROC graph. False positive rate (*fp rate*) is: 
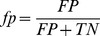



An ROC curve is created by plotting tp rate vs. fp rate at various threshold settings. As ROC curves decouple classifier performance from class skew and error cost, they have advantages over other evaluation metrics such as precision-recall curves and lift curves [27]. So AUC is often used as a measure of quality of a probabilistic classifier. In this paper, it can be used to quantify the overall ability of the model to discriminate between those author pairs who have new collaborations and those who do not.

### Feature selection

Wrapper method was used to select the most effective features from our feature vectors. Wrapper method uses a subset evaluator to create all possible subsets from the feature vector. Then it uses a classification algorithm (such as LR and SVM in this paper) to induce a classifier from the features in each subset. It will consider the subset of features with which the classification algorithm performs the best. To find a subset, the evaluator will use a search technique (such as random search, breadth first search, depth first search, and hybrid search). In this paper, breadth first search was used.

### Data source

We confined our data to the biomedical research domain, and chose “coronary artery disease” as an empirical analysis. Coronary artery disease (CAD) is the most common cause of death in the world. An estimated 17.3 million people died from cardiovascular diseases in 2008, representing 30% of all global deaths. Of these deaths, an estimated 7.3 million were due to CAD.

Co-author Qi Yu, supported by Shanxi Medical University, joined Ying Ding's research team as a visiting scholar at the Department of Library and Information Science at Indiana University (IU), USA. During his stay at IU, which lasted January 15th, 2013 through January 14th, 2014, he collected all the data used in this paper. Web of Science (WoS) was used as a data source to download CAD-related records. WoS consists of rich information for publications, including authors, publications, titles, references and so on. All of the documents containing the word “coronary” in their title, abstract or keywords were collected. These include article, meeting abstract, proceedings paper, review, editorial material, book review, letter, note, etc. The scope was limited to the years 2008 through 2013. Under the these constraints, 125,674 CAD-related documents were found.

Author name ambiguity exists in the raw data. Numerous models for author name disambiguation have been proposed within bibliographic databases and on the web. Many of these models share the broad outlines of predictive machine learning [28]. Since WoS provides full name and address for each author, a simple 2-step procedure was used in this paper to generate the disambiguated author set. First, each author's affiliation was extracted from the address. Then, the affiliation was combined with the author's full name to create a unique identifier. Of the 125,764 downloaded records, 112,324 contained address information. After name disambiguation, 425,866 authors were collected. All the results in this paper, unless otherwise specified, were calculated based on the disambiguated author set. Although the criteria we chose here are able to disambiguate the vast majority of author names, it is not sufficient, as some authors may change their affiliation or surname (due to marriage, for example) during the period under study. Therefore, future studies should strive to identify even better solutions to this problem.

Two time periods were considered for the networks: T1 = [2008–2010], and T2 = [2011–2013]. T1 was used for extracting topological features, while T2 was used for setting the corresponding labels (i.e., whether or not there was indeed a new co-author link in T2 between two authors). It is possible that some authors were only active during T1 but stopped publishing soon thereafter, or that other authors only started publishing during T2 and had been inactive during T1. To eliminate this bias, we confined the authors to those active in both time periods. The number of the resulting authors is 51,555, while 55813 authors were found active in both time periods before author name disambiguation.

We confined author pairs to those who did not co-author in the first time period but had a new co-author relationship in the second time period. We also only took into account those pairs that were 2-hop co-authors, i.e., the two authors had no less than one common co-author. Under these constraints, we first found all author pairs that have a new link in second period, and used these links as positive training pairs. 137,219 new links were found in the second time period, 3.6% of all the possible links (Table 2). Then, we sampled an equal-sized set of negative pairs so that the size of positive and negative pairs sets were balanced (274,438 pairs in total). All these author pairs, the topological features between them, and their corresponding labels comprise the entire topological feature set, on which we built our co-author relationship prediction models. We also needed another topological feature set to serve as a baseline for comparison. Thus, we randomly labelled half of the 274,438 author pairs above as “positive” and the remaining half as “negative.” All the author pairs, the topological features between them and the randomly sampled labels comprised a new topological feature set that we called “baseline topological feature set.”

**Table 2:** The summarization of the author sets with different productivity.

Author Type	# Authors	# New Relationship	# All Possible Relationship
All authors	51,555	137,219	3,838,391
# Papers > = 5	7,606	100,335	2,608,004
# Papers > = 10	2,435	64,098	1,529,799
# Papers > = 25	394	19,839	467,493
# Papers > = 50	75	5,285	117,029
# Papers > = 100	9	593	15,821

All of the documents containing the word “coronary” in their titles, abstracts or keywords were collected from Web of Science. The scope was limited to the years 2008 through 2013. Two time periods were considered for the networks: T1 = [2008–2010], T2 = [2011–2013]. The authors were confined to those acitve in both T1 and T2 periods.

We also want to know whether our model predicts collaboration relationships differently for high productive authors and less productive authors. To this end, we used five author sets: authors with no less than 5 papers, authors with no less than 10 papers, authors with no less than 25 papers, authors with no less than 50 papers, and authors with no less than 100 papers (Table 2).

## Results

### Overall accuracy

We first compared the test results from the LR model and SVM model for the entire dataset. As shown in Table 3, both LR model and SVM model scored well for all the four evaluation measures. SVM model beat LR model in terms of 3 evaluation measures: precision rate (0.697 vs. 0.696), recall rate (0.678 vs. 0.677) and AUC (0.743 vs. 0.742). This demonstrates that both models fit our data well. They were able to predict at least 67.7% of future collaborations and both performed well in discriminating between those author pairs who have new collaborations and those who do not. The AUC results outperformed those found by [10] and [18], in which co-author relationship was predicted by using a single topological feature. This means that combining topological features can yield good prediction results.

**Table 3:** Test results of LR and SVM model for entire topological feature set vs. baseline topological feature set.

Evaluation Measure	Entire topological feature set		Baseline topological feature set	
	*LR*	*SVM*	*LR*	*SVM*
Precision	0.696	0.697	0.504	0.495
Recall	0.677	0.678	0.509	0.509
F1 score	0.671	0.671	0.361	0.345
AUC	0.742	0.743	0.502	0.501

We also noted that the models built on the entire topological feature set significantly outperformed those built on the baseline topological feature set (Table 3), which means that our results were significantly better than those corresponding to normal levels.

23,594 authors published no less than 5 papers between the years 2008 and 2013. We made a prediction about the possible future links for these authors with theweights learned by the LR model. The results showed that 15,334 new co-author links will appear in the future.

### Accuracy rates for different author sets

**Figure 1:**
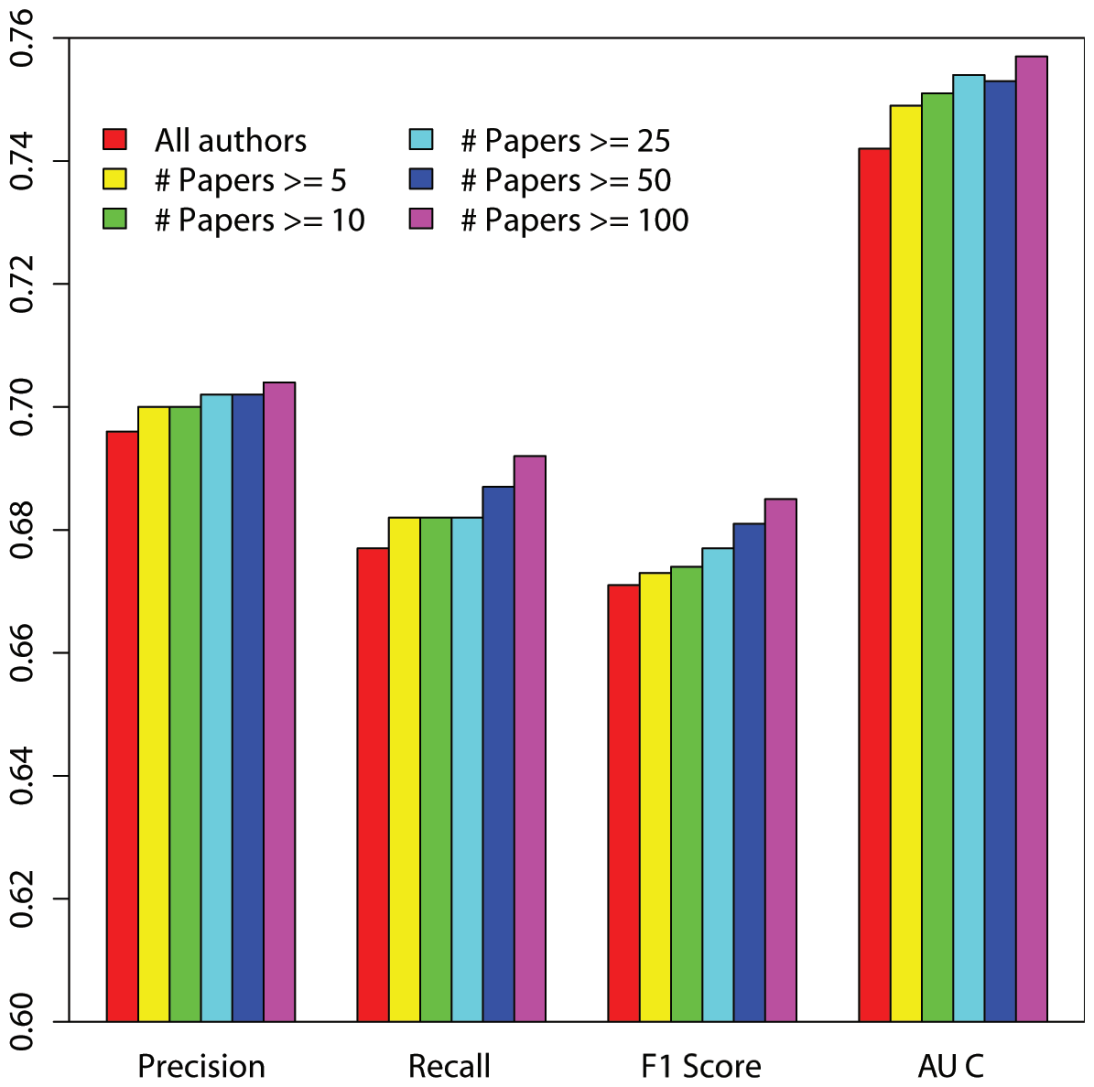
Test results of the LR model (Authors with high productivity and less productivity).

**Figure 2:**
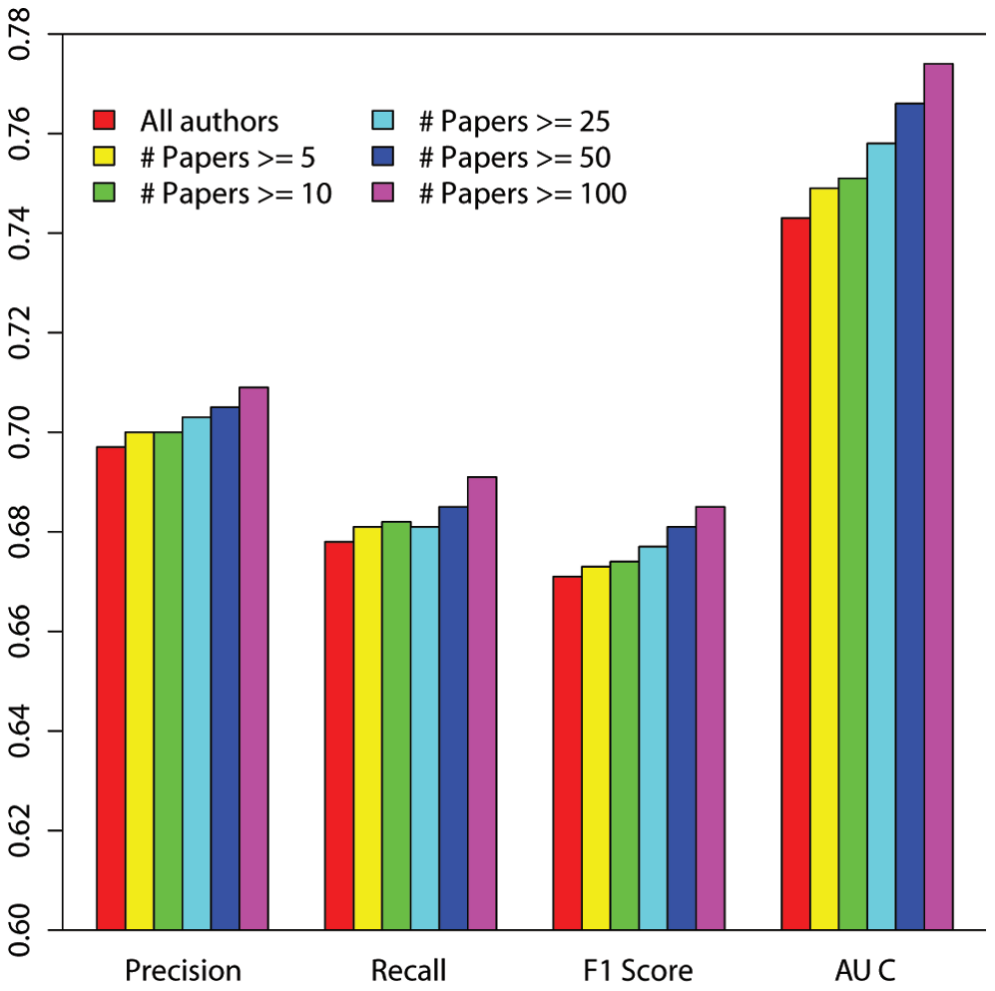
Test results of the SVM model (Authors with high productivity and less productivity).

We then compared the test results of both models for different author sets (authors with high productivity and authors with less productivity). As shown in Figure 1 and Figure 2, both learning models generally scored high for high productive author sets in terms of all the four evaluation measures, but scored low for less productive author sets. This means that both models had good ability to correctly separate the high productivity author pairs with new collaborations from those without new collaborations, similar to the results found by [10]. However, these results could be influenced by author name ambiguity, since different results were indeed found in this study before author name disambiguation: for both learning models, the precision and recall rates for highly productive authors were lower than those for less productive authors, while the AUC values for highly productive authors were higher than those for less productive authors.

### Feature selection

By using the feature selection methods mentioned above, Adamic/Adar, Preferential attachment, Katz_β_, and PropFlow were selected as the most effective ones for the LR model, while Adamic/Adar, Common Neighbors, Preferential attachment, and PropFlow were selected for the SVM model. We trained the two models with the selected features on the entire author set, and found that the testing results were improved for both the LR model and SVM model, especially for SVM model, whose AUC increased by 1.1% (Table 4). We also found that SVM model slightly outperformed LR model in terms of all the four evaluation measures.

**Table 4:** Test results of LR and SVM before vs. after using the selected topological features.

Evaluation Measure	Before using the selected topological features		After using the selected topological features	
	*LR*	*SVM*	*LR*	*SVM*
Precision	0.696	0.697	0.697	0.702
Recall	0.677	0.678	0.678	0.679
F1 score	0.671	0.671	0.671	0.672
AUC	0.742	0.743	0.744	0.754

By using the feature selection methods, Adamic/Adar, Preferential attachment, Katzβ, and PropFlow were selected as the most effective ones for boththe LR model, while Adamic/Adar, Common Neighbors, Preferential attachment, and PropFlow were selected for the SVM model.

### Individual topological feature

**Figure 3:**
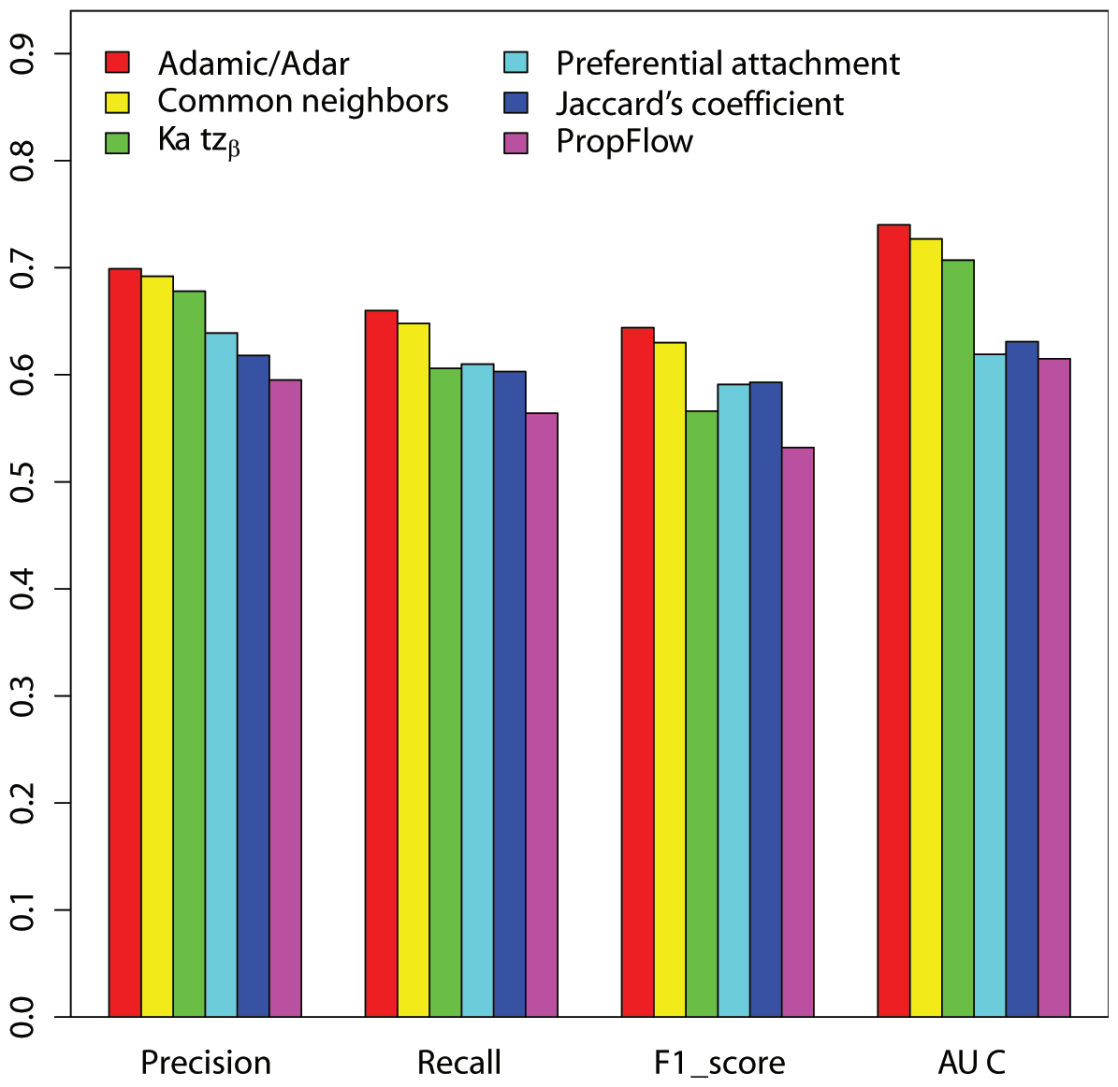
Test results of the LR model for each topological feature.

We also trained the LR model and SVM model on the entire author set by using each topological feature separately (LR model and SVM model actually produced the same test results, so we only presented the results for LR model here). As shown in Figure 3, the LR model generally produced relatively lower accuracy rates when testing topological features separately than it did when testing all the topological features as a whole. However, some features (such as Adamic/Adar, precision 0.699, recall 0.66, F1 score 0.644 and AUC 0.74) still received high evaluation scores. Surprisingly, PropFlow got a lower AUC score than topological features such as Adamic/Adar, Common Neighbor, and Jaccard's coefficient, since [18] found the opposite. Moreover, its precision, recall and F1 score were also lower than the results generated from Facebook social network data [29].

## Discussion

Firstly and foremost, both the traditionally used algorithm LR and increasingly promising algorithm SVM model performed well in co-author relationship prediction. The prediction accuracy rates as measured by the precision value, the recall value, the F1 score and AUC value, were respectively 0.696, 0.677, 0.671 and 0.742 for the LR model, and respectively 0.697, 0.678, 0.671 and 0.743 respectively for the SVM model. This is encouraging, as our predictions were solely built on topological information from the co-authorship networks, without taking into account any node-specific properties (such as geographical locations, affiliations, research topic, etc.). The reason for high prediction accuracy rates may be explained by the fact that collaboration always emerges from social networks. For instance, the “small world” phenomenon has been observed to hold true with respect to research collaboration: two scientists are more likely to collaborate or co-author a paper if they have a co-author in common. Moreover, social networks can be expanded through both informal communication such as chance encounters and formal communication such as meetings. Two authors sharing more co-authors will undoubtedly improve their chance of being acquainted with and subsequently collaborating with each other. Also, the shorter the path between two authors in a co-authorship network, the more likely they will co-author a paper in the future. In other words, the neighborhood-based and path-based topological features in co-authorship networks actually represent one or more latent features such as geographic, sub-topic and psycho-social distances. This demonstrates that co-author relationships can be predicted with high accuracy by using topological features.

Secondly, the collaborations for highly productive authors were easier to predict than less productive authors in terms of all the four evaluation measures, which means that the probability for collaborations between two authors may be affected by author productivity. This is because a highly productive author always has more neighbors than a less productive one, which can improve his visibility so that other authors are able to “find” and ultimately collaborate with him more easily. On the other hand, less productive authors always have fewer co-authors, so they are more limited in their choice of collaborators due to multiple random factors.

Thirdly, the results of feature selection showed that although the most effective features for LR model were different from those for SVM model, the testing results for both models were improved. As the SVM model beat the LR model after feature selection, the four features selected for SVM model may be the best choice for co-author relationships prediction within this dataset. Whether the same results could be achieved in other co-authorship networks remains to be seen.

Finally, when testing the topological features separately, the accuracy rates of the LR model dropped. This result is not surprising since the estimates of our LR model were affected by all the topological features. For more reliable estimates, one must include all these features. This is because omited variables in logistic regression affect coefficients through other mechanisms that operate regardless of whether omitted variables are correlated to the independent variables [30]. However, Adamic/Adar still performed well (precision 0.699, recall 0.66, F1 score 0.644 and AUC 0.74), which verifies its robustness. So Adamic/Adar can be classified as a good indicator for predicting possible co-author relationship. Furthermore, PropFlow did not perform well in our study. One possible explanation is that PropFlow may be more suitable for real networks such as the phone and Facebook networks used in [18] [29] and less so for bibliometric networks (co-authorship networks). This is supported by results generated from co-authorship network data in [29] that also received a low score for precision, recall and F1 score. We will further examine PropFlow's applicability to co-authorship networks in the future.

Overall, the methods used in our paper could be very effective in building accurate link predictors in co-authorship networks. Since the methods rely solely on topological features of the underlying networks and on general supervised learning algorithms, it can be easily applied to other networks in which link prediction is desirable.

## Conclusions

This paper presented supervised machine learning methods for building link prediction models from topological features of node pairs in co-authorship networks. The models could be useful in identifying unrealized yet potentially successful collaborations, which would in turn facilitate the development of strong research groups. In addition, we gained valuable information about which topological features are most informative for the link prediction problem, and this knowledge can be used as a basis for developing a vocabulary that supports standardized descriptions of this expertise.

However, we should also bear in mind that co-authorship is not the same as collaboration. Not every research collaboration will necessarily lead to a co-authored publication, nor all co-authored papers are results of a collaborative research process. Moreover, not all collaborators will appear as co-authors. Therefore, co-authorship is only a partial indicator of research collaboration, and we should not assume collaboration exists between two authors even if there is a co-author link between them.

There are many directions that future research in this field might take. An important next step would be testing link prediction methods in heterogeneous bibliographic network, in which there can be several types of nodes (e.g., authors and papers), and several types of links (such as write/written and cite/cited). Since a heterogeneous bibliographic network can provide more topological features to be examined for author pairs, a stronger link prediction model is likely to be obtained. Topological features are affected by name ambiguity [31] and hyperauthorship [32], two problems that pose new, open questions and directions that would be worth exploring by our research team in the future.
